# Association between intimate partner violence and the use of maternal health care services among married Malawian women

**DOI:** 10.1186/s12905-021-01312-6

**Published:** 2021-04-23

**Authors:** Praise W. Magombo, Peter A. M. Ntenda, Owen Nkoka

**Affiliations:** 1 Kamuzu Central Hospital, P.O Box 149, Lilongwe, Malawi; 2 Malaria Alert Centre (MAC), College of Medicine (CoM), University of Malawi (UNIMA), Private Bag 360, Chichiri, Blantyre 3, Malawi; 3 Institude of Health and Wellbeing, University of Glasgow, Glasgow, UK

**Keywords:** Intimate partner violence, Institutional deliveries, Skilled assistants at birth

## Abstract

**Background:**

Maternal and child health care (MCH) services aim at improving the overall health outcomes of both the mother and newborn. Intimate partner violence (IPV) has been linked with poor health outcomes and under usage of MCH services. In Malawi, IPV is a persistent problem, while MCH services’ uptake remains a constant challenge. However, there is limited information on the association between IPV and MCH services in Malawi. The study examined the association between IPV and the use of MCH services among married Malawian women.

**Methods:**

The 2015–16 Malawi demographic and health survey was used to analyze the association of IPV and the use of MCH services among 2712 married Malawian women. Multivariable logistic regression models were used to estimate the strength of association.

**Results:**

Approximately 41.4% of the women reported experiencing IPV. Specifically, 27.8%, 19.3%, and 23.6% reported experiencing physical, sexual, and emotional violence, respectively. Women who reported experiencing any form of IPV had a 34% reduced likelihood of delivering at a health facility [adjusted odds ratio (aOR): 0.66; 95% confidence interval (CI) 0.46–0.96] or were 36% less likely [aOR: 0.64; 95% CI 0.46–0.90] to have had skilled assistance during delivery compared to those who never experienced IPV.

**Conclusion:**

IPV was associated with MCH services use, specifically delivery at a health facility and skilled birth attendants. The high prevalence of IPV underscores the need to design effective programs to raise awareness regarding IPV and reduce IPV. Reducing IPV may be a promising means to support a more integrated and sustainable approach to improve the use of MCH services.

## Background

Maternal and child health (MCH) care services [e.g., antenatal care (ANC) and postnatal care (PNC)] are essential to the survival and health of both the mother and newborn [1, 2]. Globally, 303,000 maternal mortalities, 904,000 neonatal deaths, and 1.02 million stillbirths occur annually. A vast majority of these mortalities (94%) occur in low-resource settings [3]. Most of these mortalities are a result of preventable causes [4]. Therefore, there is room for designing interventions that would obviate these preventable mortalities, and MCH services provide such an opportunity.

Previous research has demonstrated several factors that are associated with the use of MCH services. For example, a study in Côte d’Ivoire revealed that women’s better income and being married were associated with maternal health services usage [5]. A systematic review in low- and middle-income countries (LMICs) cited household wealth, media exposure, and residence area as factors associated with maternal health services use among adolescent mothers [6]. One important factor linked to the reduced likelihood of using MCH services is intimate partner violence (IPV). IPV refers to any behavior within an intimate relationship that causes physical, psychological or sexual harm to those in the relationship [7]. IPV is one of the most rampant forms of violence against women, and occurs in all settings and among all socioeconomic, religious and cultural groups [7]**.** IPV exposes women to several health problems that may influence health outcomes including maternal deaths [8]. IPV affects not only the mother’s health but also the unborn child. It increases the risk of pregnancy complications and trauma such as placenta abruption [9]. IPV has severe consequences on other developmental initiatives such that the United Nations has acknowledged that ending IPV is a prerequisite for achieving sustainable development goals [10]. The World Health Organization (WHO) multi-country study on women’s health and domestic violence recognized all IPV types, with the prevalence varying from 15% in Ethiopia to 71% in Japan [11].

Although previous studies have demonstrated the association between IPV and MCH services use, there is sparse research linking IPV and MCH services utilization in Malawi. Additionally, inconsistent findings were reported among those studies that examined the association between IPV and MCH services utilization. For instance, a Bangladesh study revealed that IPV was associated with a reduced likelihood of using skilled assistance at birth [12]. Similarly, the acceptability of IPV against women was associated with a reduced likelihood of delivering at a health facility in LMICs [13]. In Zimbabwe, IPV was associated with late antenatal care booking [14]. Further, a scoping review in LMICs revealed that IPV was negatively associated with antenatal care initiation, number of visits or use of a skilled provider. Although the studies mentioned above have demonstrated the link between IPV and the use of MCH services, studies in Uganda [15] and Ethiopia [16] revealed no significant association between IPV and some aspects of MCH services such as skilled delivery care. Therefore, there is a need for setting-specific studies to be conducted as the associations may be different due to social-cultural differences. Setting-specific research may help formulate setting-specific evidence-based programs that aim to improve MCH services use and MCH outcomes.

Like any other developing country, Malawi faces immense maternal health challenges, with its maternal mortality ratio (MMR) being among the highest in sub-Saharan Africa at 634 per 100,000 live births in 2015 [17]. Several reasons could be attributed to the high MMR in Malawi, such as the non-availability of essential drugs, consumables or equipment, and lack of skilled personnel [18] that all point to a weak health system. In terms of IPV related issues, there are different kinds of domestic violence and previous research in Malawi has shown that IPV is the most common type of domestic violence [19].

This cross-sectional study assessed the association between IPV and the use of MCH services among Malawian women who were married/in a union at the survey time. It was hypothesized that women who experience IPV would be less likely to have adequate ANC, report for their first ANC on time, have institutional deliveries, use skilled assistance at birth, or have PNC within 2 days after delivery.

## Methods

### Study design and data source

This cross-sectional study used the 2015–16 Malawi Demographic and Health Survey (MDHS). The MDHS collected data from 19 October 2015 to 17 February 2016 using a two-stage stratified sampling method. The first stage involved selecting standard enumeration areas (SEAs) from the 2008 Malawi Population and Housing Census (i.e., sampling frame) and household listing from those SEAs. The second stage involved the selection of households from the sampled SEAs using equal probability systematic selection. The current study was restricted to women in a union (married or living with a partner) at the time of the survey, who had a live birth in the 5 years preceding the survey, whose partner was interviewed, and who had complete information.

### Main independent variables

The main independent variable was IPV, calculated using a total of 13 items. The 13 items fell under three categories of physical, sexual, and emotional violence. “Appendix 1” shows the specific 13 items that were assessed. A respondent reported to have ever experienced any of the forms of violence under each type of violence (i.e., at least in one type of violence under the 13 items as seen in “Appendix 1”) was coded as having IPV; otherwise, no violence. This categorization was defined as the overall IPV. In addition to the composite variable, physical, sexual, and emotional violence were also considered and assessed as separate independent variables.

### Dependent variables

The study considered five key maternal health care services as dependent variables relating to the most recent birth in the previous 5 years before the survey, namely; the number of ANC visits, the timing for first ANC, place of delivery (or institutional delivery), skilled assistance at delivery, and PNC within 2 days of delivery. The use of these services was self-reported by the participants.

### ANC visits

According to WHO recommendations, the number of ANC visits was categorized into two levels: adequate (8 or more) and inadequate (fewer than 8) [20].

### Timing for first ANC

The timing of first ANC visit was categorized as early (first trimester), and late (second/third trimester) [21].

### Institutional deliveries

Institutional deliveries defined as births that occurred at any level of health facility (i.e., government hospital/government health centre/government health post or outreach/ another public sector); private sector (private hospital or clinic/Christian Health Association of Malawi (CHAM) or Mission hospital/CHAM or Mission health centre/Banja La Mtsogolo/other private medical sectors) [22]. Those that delivered at home were regarded having were defined as “home deliveries”.

### Skilled assistance during delivery

A delivery was said to have had occurred with skilled assistance if the birth was assisted by doctors, clinical officers, medical assistants, nurses, and midwives, while those delivered through the help of traditional birth attendants (TBAs), relative/friends were defined as unskilled assistance [22].

### PNC health check within 2 days of delivery

PNC health check for the woman within 2 days after delivery by skilled professionals (skilled professionals as described above) [22].

### Covariates

Based on their importance in the literature on the outcomes under consideration [5, 6, 12], the following variables were included in the analysis: woman’s age in years (15–24, 25–34, and ≥ 35), woman’s educational level (no formal education, primary, and secondary and tertiary), household wealth calculated using principal component analysis by scoring household items and aggregating the scores into rich (upper 40%), middle (middle 20%) and poor (lower 40%), region (northern, central, and southern), residence (urban or rural), media exposure (those reporting to read newspapers, watch television, and listen to radio at least once a week categorized as yes otherwise, no), perceived distance to health facility (problem vs no problem), partner age in years (15–29, 30–44, and ≥ 45), and partner’s educational level (no formal education, primary, and secondary and tertiary).

### Statistical analysis

Weighted frequencies and percentages were presented for the study characteristics. Multivariable logistic regression models were used to test the association between IPV and selected maternal health care services using the “svy” command to account for sampling weights and survey design. Adjusted odds ratios (aOR) and 95% confidence intervals (CIs) with a significance level set at *p* < 0.05 were reported. All analyses were performed using Stata version 15 (Stata Corp, College Station, TX, USA).

### Ethical consideration

The DHS measure program provided approval to use the 2015–16 MDHS data, and the data is publicly available on their website http://dhsprogram.com/data/available-datasets.cfm. The MDHS survey protocol was approved by the Malawi National Health Sciences Research Committee and ICF Macro institutional review board. Before each interview was conducted, a verbal informed consent was sought by each interviewer reading a prescribed statement to the respondent and recording in the questionnaire whether or not the respondent consented (or provided assent on behalf of minors). Then the interviewer signed his or her name attesting to the fact that he/she read the consent statement to the respondent. The method of collecting consent has been standardized in the DHS survey to ensure consistency as some participants are not able to write.

## Results

### Population characteristics

**Fig. 1:**
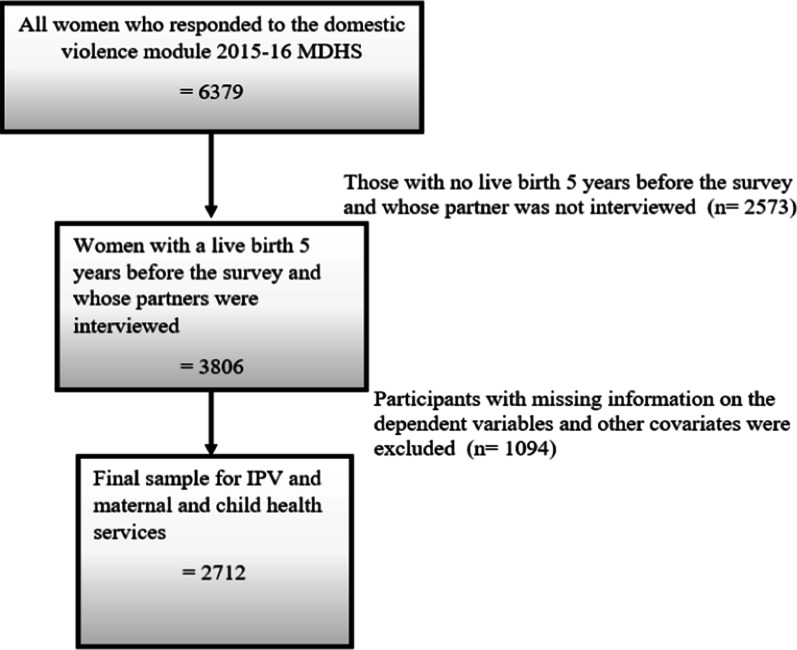
Flow chart of the sample inclusion and exclusion criteria

Table 1 presents the descriptive characteristics of the study population. A total of 2712 women were analyzed (Fig. 1 displays the sample selection process). Among others, a high proportion of the participants were aged 24–34 years (45.8%), had primary education (66.1%), from poor households (43.5%), from rural areas (86.4%), and were not exposed to media (60.7%).

**Table 1:** Characteristics of study participants

Variable	n (%)	95% CI
Woman’s age (years)		
15–24	983 (36.2)	33.7–38.8
25–34	1242 (45.8)	43.4–48.2
≥ 35	487 (18.0)	
Woman’s educational level		
No formal education	342 (12.6)	11.1 – 14.4
Primary	1793 (66.1)	63.8 – 68.3
Secondary and higher	577 (21.3)	19.2 – 23.4
Household wealth		
Poor	1180 (43.5)	40.7–46.3
Middle	544 (20.1)	18.2–22.0
Rich	988 (36.4)	33.5–39.4
Region		
Northern	336 (12.4)	10.5–14.6
Central	1273 (46.9)	43.8–50.1
Southern	1103 (40.7)	37.9–43.5
Residence		
Urban	370 (13.6)	11.0–16.7
Rural	2342 (86.4)	86.4–89.0
Media exposure		
No	1647 (60.7)	58.2–63.2
Yes	1065 (39.3)	39.3–41.8
Perceived distance to HF		
No problem	1171 (43.2)	40.3–46.1
Problem	1540 (56.8)	53.8–59.7
Partner age		
15–29	989 (36.4)	34.2–38.8
30–44	1482 (54.7)	52.2–57.1
≥ 45	241 (8.9)	7.7–10.3
Partner education		
No formal education	216 (8.0)	6.6–9.5
Primary	1617 (59.6)	57.2–62.1
Secondary and tertiary	879 (32.4)	29.9–35.0

*CI* confidence interval, *HF* health facility

### Prevalence of intimate partner violence

**Fig. 2:**
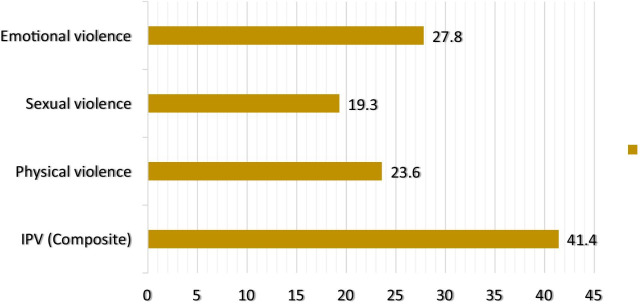
Prevalence of intimate partner violence in Malawi

Overall, approximately 41.4% of the women reported experiencing IPV. Specifically, 27.8%, 19.3%, and 23.6% reported experiencing emotional, sexual, and physical violence, respectively (Fig. 2).

### Prevalence of maternal health care services

**Fig. 3:**
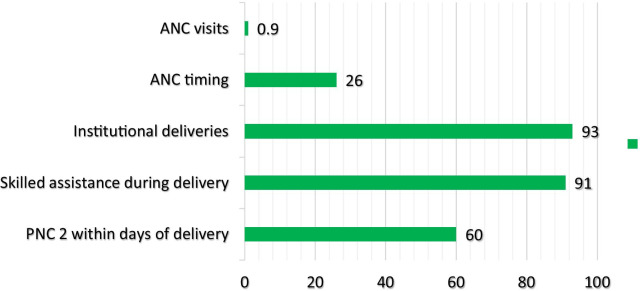
Rates of maternal health care utilization

Figure 3 displays the prevalence of several outcomes under study. Specifically, only 0.9% of the participants had adequate ANC visits (i.e., 8 or more visits) during their last pregnancy, while 26.0% reported their first ANC early. In terms of institutional deliveries, 93.0% had delivered at health facilities while skilled professionals assisted 91.0% during delivery. Among those that had skilled assistance during delivery, 99.4% delivered at a health facility. Only 60.0% of the participants had a PNC health check within 2 days after delivery.

### Association between IPV and maternal health care services utilization

The unadjusted odds ratios for the association between IPV and health care services utilization are displayed in “Appendix 2”. The multivariable analysis revealed no significant association between specific types of IPV and the outcomes under study. However, significant association were observed with the overall IPV and institutional delivery and skilled assistance at birth. Women who reported experiencing any form of violence had a 34% [aOR: 0.66; 95% CI 0.46–0.96] reduced likelihood of delivering at a health facility compared to those who did not experience any IPV. Similarly, those that reported having experienced IPV were less likely [aOR: 0.64; 95% CI 0.46–0.90] to have had skilled assistance during delivery compared to those who never experienced IPV (Table 2).

**Table 2:** Association of intimate partner violence and maternal health care services

	ANC visits	ANC timing	Institutional deliveries	Skilled assistance during delivery	PNC within 2 days after delivery
Variables	aOR (95% CI)	aOR (95% CI)	aOR (95% CI)	aOR (95% CI)	aOR (95% CI)
Physical violence^a^	0.75 (0.19–2.84)	1.04 (0.79–1.36)	0.73 (0.46–1.18)	0.79 (0.52–1.19)	0.89 (0.63–1.25)
Sexual violence^b^	–^e^	0.77 (0.56–1.06)	1.08 (0.64–1.82)	0.92 (0.59–1.45)	1.12 (0.75–1.66)
Emotional violence^c^	0.26 (0.04–1.60)	1.03 (0.78–1.37)	0.76 (0.50–1.14)	0.72 (0.49–1.07)	1.07 (0.78–1.49)
IPV (composite)^d^	0.32 (0.08–1.30)	0.89 (0.65–1.22)	**0.66 (0.46**–**0.96)**	**0.64 (0.46**–**0.90)**	0.92 (0.69–1.24)

Models were adjusted for woman’s age, women’s educational level, parity, household wealth, region, residence, media exposure, distance to nearest health facility, partner age, and partner educational level

*IPV* intimate partner violence, *aOR* adjusted odds ratio, *CI* confidence interval, *ANC* antenatal care, *PNC* postnatal care

^a^Reference; no physical violence

^b^Reference; no sexual violence

^c^Reference; no emotional violence

^d^Reference; no any violence experienced

^e^There were no samples in those who experienced sexual violence and those that had adequate ANC visits

## Discussion

The study assessed the association between IPV and the use of MCH services among married Malawian women. According to our literature review, this is the first study to examine this association in Malawi. Findings from the current research show evidence of an association between overall IPV and maternal health care services utilization.

Women who experienced IPV were less likely to deliver at a health facility than those who reported not to have experienced IPV. Women in an abusive relationship may be afraid to leave their abusive relationships, exhibit low self-esteem and insufficient autonomy [23] which may influence their health care decisions, thereby affecting their use of maternal health care services. Better women’s autonomy on household decision making was associated with an increase in the odds of delivering at a health facility in LMICs [13]. Additionally, women who experience IPV may resign to their fate, lose self-esteem and confidence. Ultimately, this may affect their use of MCH services, as observed in a study conducted in LMICs [13]. Our results suggest that Malawian women may be dependent on their spouses’ decision-making regarding treatment-seeking; if women experience IPV or are in abusive relationships, they may be less likely to visit health facilities for help.

Consistent with a study conducted in Mozambique, women who reported having experienced IPV were more likely to be assisted by family members, traditional healers, and traditional birth attendants than skilled attendants [24]. Similar results were found in a study from Nigeria, particularly among women who had been emotionally abused [25]. Additionally, in Bangladesh, similar results were reported, with women reported to have experienced IPV being less likely to deliver their babies with assistance from skilled professionals [12]. IPV has been associated with stress and depressive symptoms [26, 27] which may lead to a demotivation among women to enhance/pursue appropriate care-seeking behaviors.

Our study findings revealed no significant association between specific forms of violence nor overall IPV with ANC visits and timing and PNC visits within 2 days of delivery. Findings from a scoping review of LMICs reported a negative association between IPV and ANC initiation and the number of ANC visits [28]. Our results may partly underline the effectiveness of community outreach programs for ANC in Malawi, such as mobile clinics and community health workers (CHWs). The community outreach programs enable women to access ANC services closer to their communities and enhance timely identification of pregnancies by the CHWs [29]. Therefore, regardless of whether women may experience IPV in these settings, they may still manage to book for ANC since these initiatives make these services easily accessible.

The prevalence of overall IPV in Malawi was 41.4% representing a rise from the 27% reported in 2004 [30]. The sharp increase in IPV may be due to strengthened reporting systems put in place coupled with increased awareness among women on IPV issues over time. Nevertheless, it is essential to note that IPV is a public health concern in Malawi, with emotional violence (27.8%) being the most reported form of violence among women in a union. Often, emotional violence may appear harmless and may easily be neglected [31]. However, the high proportion of women reporting emotional violence in the current study underscores the importance of incorporating emotional violence messages in awareness IPV campaigns in Malawi.

### Policy/program implications

The findings have policy/program implementations in Malawi:

1. Programs aimed at improving maternal and child health should be integrated with gender-based violence programs for effective implementation.
2. Findings from the analysis revealed no significant association between specific types of IPV and MCH services, suggesting that IPV should be tackled in its entirety in Malawi.
3. In areas where IPV is high, it may be essential to sensitize, collaborate, and build the capacity of TBAs to provide quality services and, further, establish robust referral systems to the mainstream health care service.

### Strengths and limitations

The study used data from a nationally representative sample; therefore, this analysis may be generalized to married Malawian women. However, the cross-sectional design of the survey could not allow causal inferences to be drawn. The timing of exposure and outcome could not be determined considering the cross-sectional nature of our data. Some women may have experienced IPV after their pregnancy. Therefore, our results should be carefully interpreted.

Additionally, due to the sensitive nature of the main independent variables, there is a possibility that underreporting may have occurred, which may have subsequently influenced the findings. This limitation may have been cured through the use of well-trained MDHS surveyors. The confirmation of confidentiality may have created a comfortable environment for the interviewees to discuss such issues related to IPV. Some of the women who responded to the violence module were not analyzed based on our inclusion criteria for analysis (i.e., have a live birth within 5 years before the survey, spouse had to be interviewed, and only those with complete information). As a result, we may have lost information from women who had given birth more than 5 years ago before the survey. However, excluding them was aimed to examine the independent variables within a reasonable time frame to avoid recall bias. Finally, there are other repercussions of IPV during and after pregnancy (e.g., depression and anxiety) that have not been assessed in the current analysis, which may help understand some of the associations observed in this study and may be critical for public health programming in Malawi. Future studies should examine the association between IPV with mental health disorders and their impact on MCH services use in Malawi.

## Conclusion

The current study has revealed that women who experienced IPV were less likely to deliver at a health facility and use skilled assistance during delivery. These two outcomes are essential maternal health services; thus, programs aimed at improving maternal and child health must be integrated with gender-based violence programs. This integration would ensure that efforts are consolidated in implementing the programs to achieve the desired outcomes (i.e., improve overall maternal and child health).

## Data Availability

The study used, with permission, data from the International Classification of Functioning, Disability, and Health (ICF). The data is publicly available upon request from the ICF on (https://dhsprogram.com/data/available-datasets.cfm).

## Appendix 1

Specifically, the following items were collected;

Physical violence:

1. pushing/shaking/throwing somethings at her,
2. slapping her,
3. twisting her arm/pulling her hair,
4. punching her with fists or hitting her with something harmful,
5. kicking/dragging/beating her,
6. choking/burning her on purpose,
7. threatening/attacking her with a weapon (such as knife),

Sexual violence:

(8) forced sexual intercourse,
(9) physically forcing her to perform any other sexual act when undesired,
(10) forcing her with threats to perform sexual acts when undesired,

emotional violence:

(11) humiliating her in public,
(12) threatening to hurt/harm someone close to her,
(13) insulting/making her feel bad about herself.

## Appendix 2

See Table

**Table 3:** Association of intimate partner violence and maternal health care services (unadjusted estimates)

	ANC visits	ANC timing	Institutional deliveries	Skilled assistance during delivery	PNC within 2 days after delivery
Variables	OR (95% CI)	OR (95% CI)	OR (95% CI)	OR (95% CI)	OR (95% CI)
Physical violence^a^	0.74 (0.18–3.01)	1.02 (0.77–1.33)	0.68 (0.42–1.10)	0.75 (0.50–1.14)	0.91 (0.64–1.29)
Sexual violence^b^	–^e^	0.79 (0.58–1.07)	0.98 (0.61–1.57)	0.85 (0.55–1.31)	1.09 (0.74–1.59)
Emotional violence^c^	0.27 (0.05–1.53)	1.03 (0.80–1.33)	0.70 (0.45–1.07)	0.68 (0.46–1.01)	1.03 (0.75–1.42)
IPV (composite) ^d^	0.32 (0.08–1.32)	0.93 (0.74–1.18)	**0.58 (0.40**–**0.85)**	**0.59 (0.42**–**0.82)**	0.94 (0.71–1.25)

*IPV* intimate partner violence, *OR* odds ratio, *CI* confidence interval, *ANC* antenatal care, *PNC* postnatal care

^a^Reference; no physical violence

^b^Reference; no sexual violence

^c^Reference; no emotional violence

^d^Reference; no any violence experienced

^e^There were no samples in those who experienced sexual violence and those that had adequate ANC visits

3.

## Abbreviations

IPV: Intimate partner violence
MMR: Maternal mortality rate
MCH: Maternal and child health
SEA: Standard enumeration area
ANC: Antenatal care
PNC: Postnatal care
SSA: Sub-Saharan Africa
aOR: Adjusted odds ratio
CI: Confidence interval
MDHS: Malawi demographic health survey
CHAM: Christian health association of Malawi

## References

[1] Addisse M. Maternal and child health care. Lecture notes for health science students. Ethiopian Public Health Training Initiative. 2003.

[2] World Health Organization. Strategies towards ending preventable maternal mortality (EPMM). 2015. https://apps.who.int/iris/bitstream/handle/10665/153544/9789241508483_eng.pdf. Assessed 20 March 2021.

[3] Centers for Disease Control and Prevention. Maternal and child health. https://www.cdc.gov/globalhealth/mch/index.htm. Accessed 27 Nov 2020.

[4] World Health Organization. Maternal mortality. https://www.who.int/news-room/fact-sheets/detail/maternal-mortality. Accessed 20 March 2021.

[5] Samba M, Attia-Konan AR, Sangaré AD, Youan GJ, Kouadio LP, Bakayoko-Ly R (2020). Factors associated with the use of maternal health services by mothers in a post-conflict area of western Côte d'Ivoire in 2016. BMC Health Serv Res.

[6] Banke-Thomas OE, Banke-Thomas AO, Ameh CA (2017). Factors influencing utilisation of maternal health services by adolescent mothers in low-and middle-income countries: a systematic review. BMC Pregnancy Childbirth.

[7] World Health Organization. Understanding and addressing violence against women. http://apps.who.int/iris/bitstream/10665/77432/1/WHO_RHR_12.36_eng.pdf. Accessed 20 March 2021.

[8] Musa A, Chojenta C, Geleto A, Loxton D (2019). The associations between intimate partner violence and maternal health care service utilization: a systematic review and meta-analysis. BMC Womens Health.

[9] Leone JM, Lane SD, Koumans EH, DeMott K, Wojtowycz MA, Jensen J, Aubry RH (2010). Effects of intimate partner violence on pregnancy trauma and placental abruption. J Womens Health.

[10] Babu BV, Kusuma YS (2017). Violence against women and girls in the sustainable development goals. Health Promot Perspect.

[11] Abramsky T, Watts CH, Garcia-Moreno C, Devries K, Kiss L, Ellsberg M, Jansen HA, Heise L (2011). What factors are associated with recent intimate partner violence? Findings from the WHO multi-country study on women's health and domestic violence. BMC Public Health.

[12] Rahman M, Nakamura K, Seino K, Kizuki M (2012). Intimate partner violence and use of reproductive health services among married women: evidence from a national Bangladeshi sample. BMC Public Health.

[13] Sripad P, Warren CE, Hindin MJ, Karra M (2019). Assessing the role of women’s autonomy and acceptability of intimate-partner violence in maternal health-care utilization in 63 low-and middle-income countries. Int J Epidemiol.

[14] Shamu S, Munjanja S, Zarowsky C, Shamu P, Temmerman M, Abrahams N (2018). Intimate partner violence, forced first sex and adverse pregnancy outcomes in a sample of Zimbabwean women accessing maternal and child health care. BMC Public Health.

[15] Kwagala B, Nankinga O, Wandera SO, Ndugga P, Kabagenyi A (2016). Empowerment, intimate partner violence and skilled birth attendance among women in rural Uganda. Reprod Health.

[16] Mohammed BH, Johnston JM, Harwell JI, Yi H, Tsang KW, Haidar JA (2017). Intimate partner violence and utilization of maternal health care services in Addis Ababa, Ethiopia. BMC Health Serv Res.

[17] UNICEF. Maternal and Newborn Health Disparities in Malawi. https://data.unicef.org/wp-content/uploads/country_profiles/Malawi/country%20profile_MWI.pdf. Accessed 20 March 2021.

[18] Mgawadere F, Unkels R, Kazembe A, van den Broek N (2017). Factors associated with maternal mortality in Malawi: application of the three delays model. BMC Pregnancy Childbirth.

[19] Pelser E, Gondwe L, Mayamba C, Mhango T, Phiri W, Burton P: Intimate partner violence: Results from a national gender-based violence study in Malawi. 2005. https://issafrica.s3.amazonaws.com/site/uploads/Book2005PartnerViolence.PDF. Accessed 27 Nov 2020.

[20] WHO. WHO recommendations on antenatal care for a positive pregnancy experience: summary. WHO; Geneva. https://www.who.int/publications/i/item/9789241549912. Accessed 20 March 2021.

[21] Nkoka O, Chuang TW, Chen YH (2018). Association between timing and number of antenatal care visits on uptake of intermittent preventive treatment for malaria during pregnancy among Malawian women. Malar J.

[22] National Statistical Office—NSO/Malawi and ICF. Malawi demographic and health survey 2015–16. Zomba, Malawi: NSO and ICF. 2017.

[23] Tenkorang EY (2018). Women's autonomy and intimate partner violence in Ghana. Int Perspect Sex Reprod Health.

[24] Tura H, Licoze A (2019). Women’s experience of intimate partner violence and uptake of Antenatal Care in Sofala, Mozambique. PLoS ONE.

[25] Ononokpono DN, Azfredrick EC (2014). Intimate partner violence and the utilization of maternal health care services in Nigeria. Health Care Women Int.

[26] Bailey BA (2010). Partner violence during pregnancy: prevalence, effects, screening, and management. Int J Women’s Health.

[27] Von Eye A, Davidson WS, Bogat GA, Levendosky AA, DeJonghe E. Pathways of suffering: the temporal effects of domestic violence on women's mental health. Maltrattamento e abuso all’infanzia. 2004.

[28] Metheny N, Stephenson R (2017). Intimate partner violence and uptake of antenatal care: a scoping review of low-and middle-income country studies. Int Perspect Sex Reprod Health.

[29] Kachimanga C, Dunbar EL, Watson S, Cundale K, Makungwa H, Wroe EB, Malindi C, Nazimera L, Palazuelos D, Drake J (2020). Increasing utilisation of perinatal services: estimating the impact of community health worker program in Neno, Malawi. BMC Pregnancy Childbirth.

[30] Hindin MJ, Kishor S, Ansara DL. Intimate partner violence among couples in 10 DHS countries: predictors and health outcomes [Internet]. Vol. No. 18, DHS Analytical Studies. Calverton, Maryland: Macro International Inc.; 2008. p. 78. https://dhsprogram.com/publications/publication-AS18-Analytical-Studies.cfm. Accessed 20 March 2021.

[31] Kaur R, Garg S (2008). Addressing domestic violence against women: an unfinished agenda. Indian J Community Med.

